# Management of Acute Fatty Liver of Pregnancy: A Retrospective Study of 12 Cases Compared With Data in the Literature

**DOI:** 10.7759/cureus.85753

**Published:** 2025-06-11

**Authors:** Outhmane Maalbi, Nabil Elachhab, Abdelmajid Elkabbaj, Manal Arfaoui, Salim Hindi, Sophia Lahbabi, Nezha Oudghiri, Rajae Tachinante

**Affiliations:** 1 Maternal Critical Care and Anesthesiology Departement, Ibn Sina University Hospital, Rabat, MAR

**Keywords:** acute fatty liver of pregnancy, bright liver, delivery, hemostasis, plasmapheresis, steatosis, swansea criteria, uterine atony

## Abstract

Introduction

Acute fatty liver of pregnancy (AFLP) is a rare and potentially life-threatening obstetric condition marked by hepatic dysfunction due to fat accumulation in liver cells. It generally arises in the later stages of pregnancy or shortly after delivery. Clinical presentation is often nonspecific, with symptoms such as gastrointestinal discomfort, nausea, and increased thirst or urination. In more severe cases, signs of liver failure may develop, including jaundice, altered mental status, and coagulation abnormalities. Laboratory tests typically reveal elevated liver enzymes, impaired coagulation, and abnormalities in blood counts. The condition poses significant risks for both mother and fetus, and timely diagnosis and appropriate multidisciplinary management are essential for favorable outcomes.

Methods

The objective of our study was to analyze the management of this pathology in our intensive care unit and compare it with the literature. This is a retrospective, descriptive, and analytical study conducted in the intensive care unit of Souissi Maternity Hospital, including 12 cases admitted from January 1, 2023, to December 31, 2024. We used a data extraction sheet covering demographic, diagnostic criteria, complications, and maternal and obstetric management, to analyze the data collected from medical records of pregnant women.

Results

Our retrospective study of 12 AFLP cases revealed a mean patient age of 29.8 ± 5.24 years and an average gestational age of 34.8 weeks. Gravidity and parity medians were 2 and 2.5, respectively. Gestational hypertension was present in five of the patients (41.7%), with some complicated by preeclampsia or eclampsia. All 12 patients met the Swansea diagnostic criteria (more than six criteria for each patient), with jaundice in 11 patients (91.7%), nausea/vomiting in nine of them (75%), and epigastric pain in seven parturients (58.3%) being the most common clinical presentations. Laboratory findings showed elevated transaminases in 10 patients (83% >3x normal, mean aspartate aminotransferase (AST) of 683.36 IU/L, mean alanine aminotransferase (ALT) of 428 IU/L), and total bilirubin was elevated >14 µmol/L in all patients, mean 169 µmol/L). Coagulopathy was common, with eight patients (66%) having a prothrombin time (PT) < 70%. Maternal complications were frequent in 11 patients (95%), including renal failure in eight of them (72%), hemorrhagic complications in five patients (45%), often necessitating blood transfusions, altered consciousness, and sepsis. Fetal complications included four intrauterine fetal death (33%) and three acute fetal distress (25%). Management was multidisciplinary, focusing on prompt uterine evacuation, hemostasis correction, and management of renal, infectious, neurological, and respiratory complications. No patients in our cohort received plasmapheresis due to equipment unavailability.

Conclusion

Both the existing literature and our service's protocol prioritize immediate fetal delivery as the definitive intervention to halt disease progression. While the literature explores adjunctive therapies such as N-acetylcysteine (NAC) and plasmapheresis, the core focus remains on meticulous supportive care to address the numerous complications arising from liver failure.

## Introduction

Acute fatty liver of pregnancy (AFLP) represents a rare yet severe obstetric emergency, sometimes referred to as acute yellow liver atrophy [1-3]. This critical condition typically manifests during the third trimester of gestation or in the immediate postpartum period [2,4]. Its clinical description dates back to Sheehan, who first observed its presentation as rapidly progressive liver failure with jaundice in the third trimester, characterized by microsteatosis, hepatocyte ballooning, and minimal necrosis or inflammation on liver biopsy [5].

The global prevalence of AFLP is estimated to range from one to three cases per 10,000 deliveries, though more contemporary studies indicate possibly lower rates, around 2% [6]. While its precise etiology remains elusive, AFLP has been consistently linked to mitochondrial dysfunction affecting fatty acid oxidation. A significant genetic factor is a deficiency in long-chain fatty acyl-CoA dehydrogenase (LCHAD), an autosomal recessive disorder, which predisposes to AFLP and can lead to acute maternal liver failure through hepatic inflammation and fibrosis [7-9].

Recognized risk factors for AFLP include a history of the condition in previous pregnancies, advanced gestational age, male fetal sex, multiple pregnancies, hypertensive disorders of pregnancy, and gestational weight gain exceeding 18 kg [10,11]. Given its potential for severe maternal and fetal complications, such as hemorrhage, multisystem organ dysfunction, and intrauterine fetal death, timely diagnosis and effective management are critically important. The Swansea criteria [12-14] are widely accepted diagnostic tools for AFLP, notable for their high sensitivity and specificity [12]. A diagnosis of AFLP requires at least six criteria to be met [11].

This study aims to expand on the understanding of AFLP by retrospectively analyzing 12 cases admitted to the maternity department of Ibn Sina Hospital, Rabat, Morocco, over a two-year period (2023-2024). We detail the clinical features, laboratory findings, and complications observed within our cohort, comparing these with established literature. Furthermore, we critically evaluate our diagnostic and therapeutic strategies to contribute to improved management protocols for this challenging obstetric complication.

## Materials and methods

Study type and location

This is a retrospective and descriptive study conducted at the referral intensive care unit (ICU) of the Souissi Maternity Hospital, which is part of the Ibn Sina University Hospital Center in Rabat, Morocco. The study period spanned two years, from January 1, 2023, to December 31, 2024.

Study population

The study cohort included 12 pregnant women diagnosed with AFLP. These patients, aged between 24 and 37 years, were admitted for delivery at our institution and received an AFLP diagnosis based on established clinical and laboratory criteria.

Inclusion criteria 

Pregnant women who were hospitalized in the intensive care unit for AFLP, with the diagnosis confirmed according to the Swansea clinical and biological criteria.

Exclusion criteria 

Patients with associated liver pathologies not directly related to AFLP were excluded from the study.

Data collection

Data were systematically extracted from the patients' medical records and recorded onto a standardized operating sheet. This sheet was completed by the department's residents and included comprehensive information about age, antecedents, risk factors, diagnostic criteria, complications, and maternal and obstetric management.

The data were analyzed descriptively and analytically, allowing us to assess risk factors, clinical characteristics, associated complications, and the effectiveness of management in our department. The results obtained were compared with data from the literature to identify possible differences in management strategies for this pathology.

## Results

Demographic characteristics

The mean age of our population is 29.8 ± 5.24 years, with extremes of ages between 24 and 37 years old. The average gestational age (GA) of parturients included in our study is 34.8 weeks of amenorrhea, the minimum GA is 26, and the maximum is 39. The median of gravidity in our study is 2 [1-3], while the median of parity is 2.5 [1-4].

Risk factors

Ten patients had multiparty, and four others had a male fetus. Furthermore, no patient had a history of AFLP in previous pregnancies or a BMI below 20.

Medical and obstetrical antecedents

Among the 12 patients included in the study, five patients (41.7%) presented with gestational hypertension (GHT) defined by SBP ≥ 140 mmHg and/or DBP ≥ 90 mmHg with no proteinuria, three of them were complicated by superimposed preeclampsia (SBP ≥ 160 mmHg and/or DBP ≥ 110 mmHg, with proteinuria > 0.3 g/24h). One patient had isolated preeclampsia, while another developed eclampsia. In addition, one patient reported a family history of hypertension. Finally, three patients (25%) had no significant medical history.

Presenting clinical signs

The most common symptom on admission was jaundice, which was found in 11 patients, a rate of 91.7%, followed by nausea and vomiting, which was found in nine patients, a rate of 75%. Furthermore, 58.3% (or seven) of the patients in our study reported epigastralgia (58.3%), and five of them had polyuria-polydipsia (41.7%). All of these symptoms are included in the Swansea criteria, and the majority of parturients in our study have them.

Laboratory findings

Transaminase testing was performed on all patients and revealed a mean aspartate aminotransferase (AST) value of 683.36 IU/L (with a range of 55-2285 IU/L) and a mean alanine aminotransferase (ALT) value of 428 IU/L (with a range of 31-1702 IU/L). In our study, 10 patients (or 83%) presented with hepatic cytolysis with transaminases elevated more than three times the normal.

Direct and indirect bilirubin were measured in all our patients, revealing a mean total bilirubin of 99 mg/L (169 µmol/L), with a range of 35-264 mg/L (59.85-451 µmol/L). All 12 patients in our study had bilirubin levels above 14 µmol/L, the threshold used in the Swansea criteria.

Regarding hemostasis assessment, a prothrombin time (PT) was performed on all patients. The mean PT value was 58%, with a range from 32% to 100%. Eight patients (or 66%) had a low PT (<70%). A platelet count was performed on our patients; the mean value was 140,833/mm^3^, with a range between 33,000 and 380,000/mm^3^.

A white blood cell count was requested for all patients; the average white blood cell count was 17,266/L, with extremes of 9,000 and 27,200/L.

Blood glucose levels were measured in all patients; the average was 75 mg/dL, with a range of 40-102 mg/dL. In our study, 50% of the patients had blood glucose levels below 72 mg/dL (<4 mmol/L).

Creatinine levels were measured in all our patients; the average was 24.4 mg/L, with a range of 6.3-49 mg/L. Seven patients (or 58.33%) presented with renal failure, with a creatinine level greater than 16.9 mg/L (or 150 µmol/L).

Serum ammonia measurement is a key element in the diagnosis of AFLP. In our study, it was requested in a single patient and returned 58 µmol/L, which corresponds to the Swansea criterion of a serum ammonia greater than 47 µmol/L.

Factor V was performed in only three patients, with rates of 21%, 66%, and 106%.

Abdominal ultrasound

**Figure 1:**
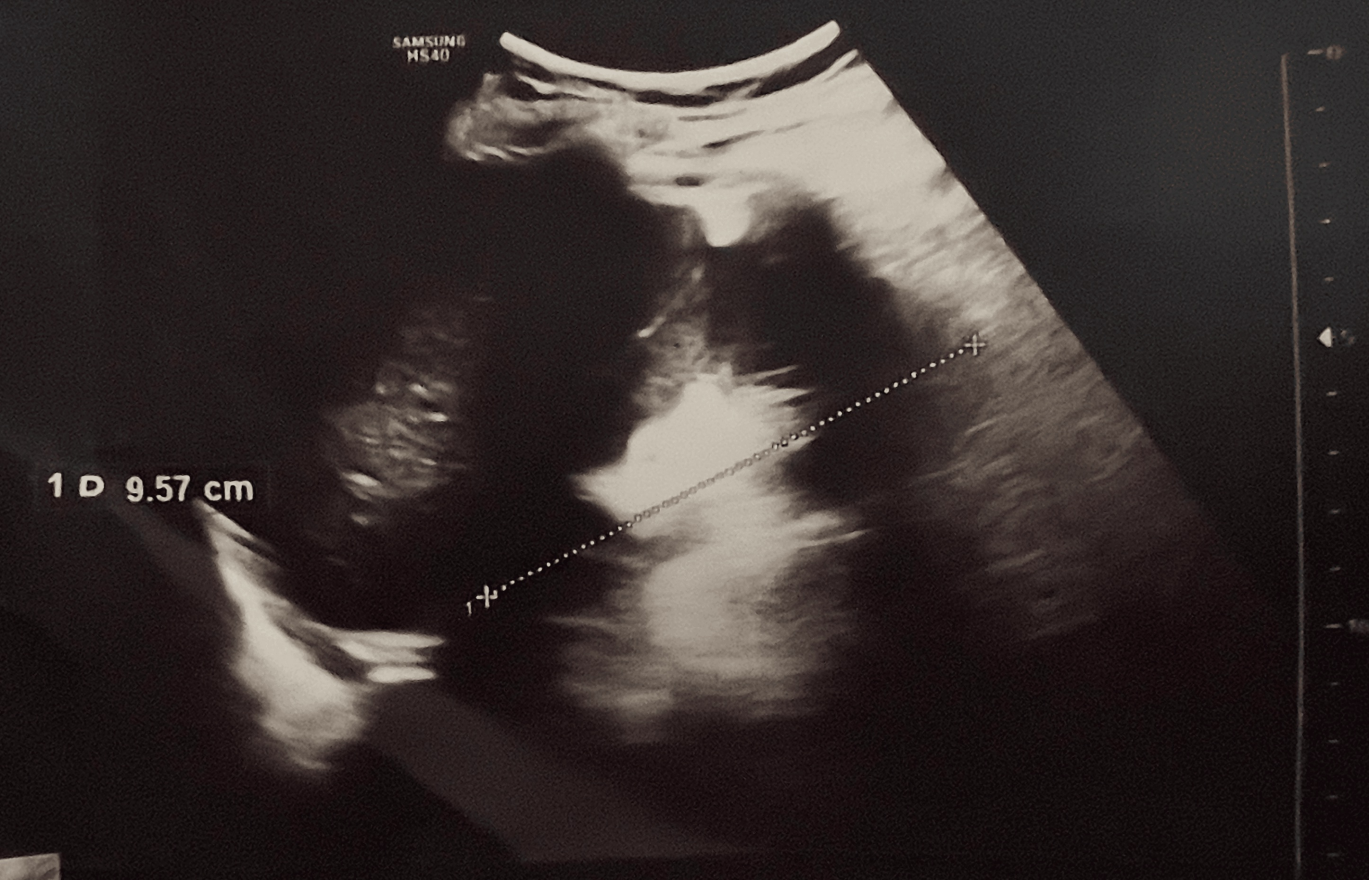
Hepatic steatosis (bright liver)

Ultrasound was performed in all patients. Five of them (41.7%) presented a mild-to-moderate ascites. Hepatic steatosis, identified by a bright liver appearance, was detected in only 25% of cases (three patients) (Figure 1). A subcapsular hepatic hematoma was observed in one case (8.3%). Additionally, the ultrasound findings were unremarkable in four patients (33%). 

Maternal complications 

**Figure 2:**
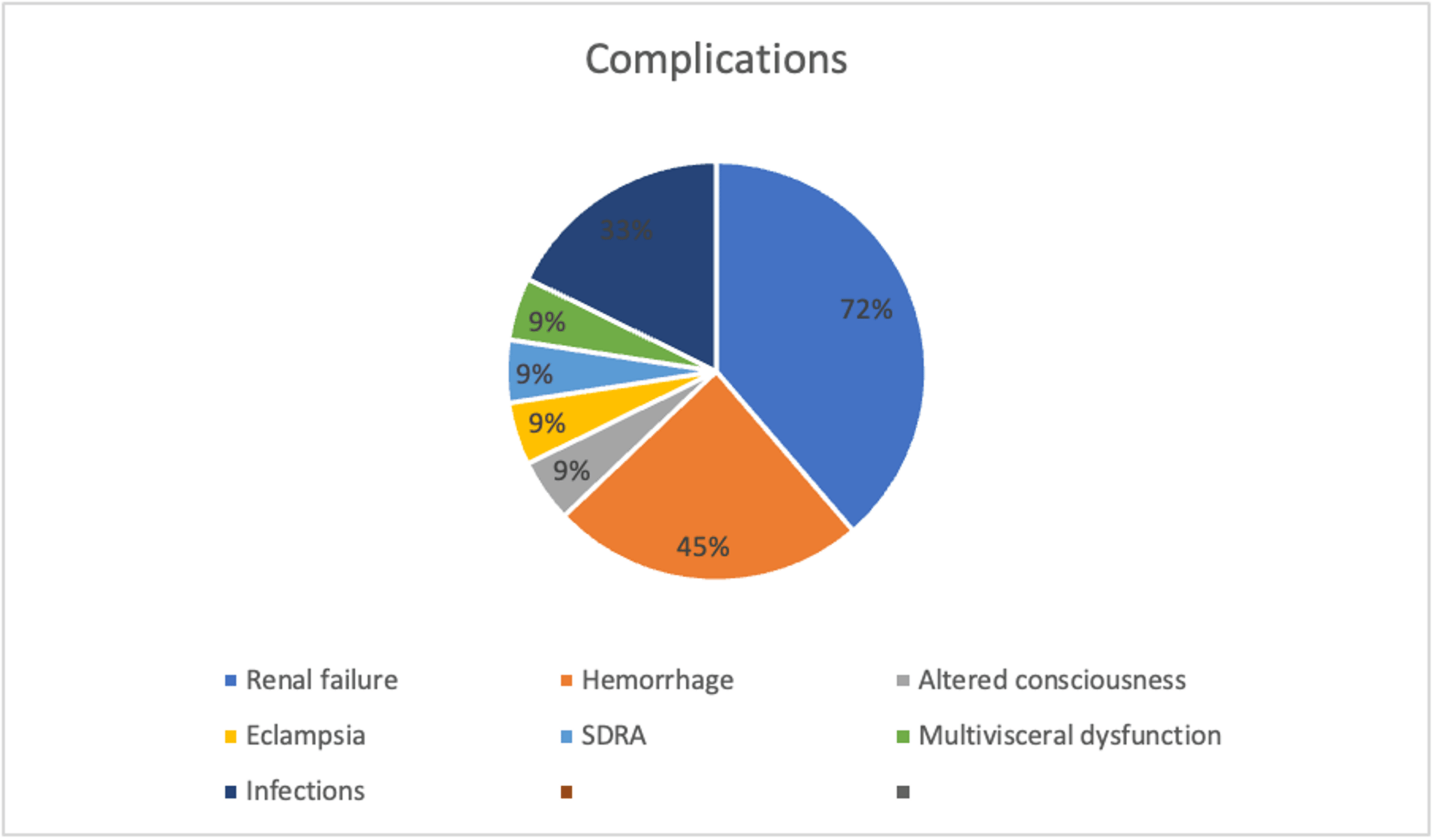
Maternal complications

A total of 95% of the parturients (i.e., 11 out of 12 patients) developed severe complications. Renal failure was the most frequent complication, occurring in 72% of cases (eight patients), followed by hemorrhagic complications reported in five patients (45%), including two cases related to uterine atony. Four patients (33%) experienced a retroplacental hematoma. One patient presented with an altered level of consciousness due to hypoglycemia. Another patient developed generalized convulsive seizures consistent with eclampsia. Additionally, one patient (9.1%) developed acute respiratory distress syndrome (ARDS) with multiorgan failure, leading to death (Figure 2).

Fetal complications 

Among the 12 births, eight newborns (66%) presented complications: four intrauterine fetal deaths (33%), three cases of acute fetal distress (25%) with low Apgar scores at birth (Apgar <7), and one case of intrauterine growth restriction (IUGR) (8%).

Management

The management was both medical and obstetrical, involving obstetricians and anesthesiologist-intensivists, and was based on three main principles: performing uterine evacuation, ensuring adequate hemostasis, and managing renal, infectious, neurological, and respiratory complications.

Initial stabilization was realized by oxygen therapy, fluid resuscitation with crystalloids (normal saline, lactated Ringer’s), correction of hypoglycemia, and antihypertensive treatment.

During their ICU stay, five patients (41%) required peripartum transfusion of labile blood products, including packed red blood cells (PRBCs), fresh frozen plasma (FFP), and platelet concentrates (PC): two patients for anemia (hemoglobin levels of 6 and 7 g/dL), one patient due to iron deficiency and the other due to hemorrhage, one patient for postpartum hemorrhage due to cervical laceration, and two patients for uterine atony.

For obstetrical management, vaginal delivery was performed in six patients (50%) in other healthcare structures and were referred to our unit. The remaining half underwent cesarean section in our hospital. Hemostatic hysterectomy was performed in the two patients with uterine atony, along with massive transfusion involving PRBCs, FFP, platelets, and fibrinogen, and the use of vasopressor agents.

Two patients (16%) underwent dialysis for stage 3 acute kidney injury according to the Kidney Disease Improving Global Outcomes (KDIGO) classification (serum creatinine ≥3 times normal with oliguria <0.3 mL/kg/h).

In our study, none of the patients received plasmapheresis due to the unavailability of appropriate equipment.

## Discussion

AFLP, also referred to as acute yellow liver atrophy, is a rare but critical obstetric emergency [1-3]. This severe condition typically presents during the third trimester of pregnancy or in the early postpartum period [2,4]. Sheehan was the first to clinically describe AFLP, detailing it as jaundice with rapidly progressive liver failure in the third trimester, characterized by distinct histological features such as microsteatosis, hepatocyte ballooning, and minimal hepatocellular necrosis or inflammation on liver biopsy [5].

The global prevalence of AFLP is estimated between one and three cases per 10,000 deliveries. However, some more recent studies suggest significantly lower rates, around 2% [6]. Our study analyzed 12 cases of AFLP hospitalized in the maternity department of Maternité Souissi Hospital over a two-year period (2023-2024). We compared our findings with existing literature to enhance diagnostic and therapeutic approaches to this condition.

The exact etiology of AFLP remains unclear, but it has been consistently linked to mitochondrial dysfunction in fatty acid oxidation. Furthermore, a deficiency in long-chain fatty acylhydroxy-CoA dehydrogenase (LCHAD), an autosomal recessive disorder, has been associated with AFLP, potentially leading to severe acute maternal liver failure by exposing the liver to inflammation and fibrosis [7-9]. Several risk factors have been identified for AFLP, including a history of AFLP in previous pregnancies, advanced GA, male fetal sex, multiple pregnancies, hypertensive disorders of pregnancy, and gestational weight gain exceeding 18 kg [10,11].

The Swansea criteria [3,12-14] are the internationally recognized diagnostic criteria for AFLP. These criteria demonstrate high diagnostic utility, with reported sensitivity of 100%, specificity of 57%, positive predictive value of 85%, and negative predictive value of 100% [3,12]. A diagnosis of AFLP requires at least six criteria to be met [3,11]. In our cohort, all 12 patients met more than six Swansea criteria. The most frequent clinical signs observed were jaundice, nausea, vomiting, polyuria-polydipsia, and epigastric pain. These findings are consistent with existing literature, where jaundice is reported in 60-100% of cases, nausea and vomiting in 50-82%, and epigastric pain in 40-78% of pregnant women [2,15,16]. The European Association for the Study of the Liver (EASL) recommendations also delineate severity criteria that warrant intensive care unit admission, including encephalopathy, elevated lactate levels, more than seven Swansea criteria, or a model for end-stage liver disease (MELD) score ≥30 [17].

Regarding laboratory findings, transaminase levels in AFLP typically rise to approximately ten times the normal range [18]. Our study confirmed this pattern, with mean AST and ALT levels found to be 10 times above normal. While bilirubin levels are often normal in the early disease course, conjugated hyperbilirubinemia usually develops as the condition progresses, rarely exceeding 170 μmol/L [18]. In our study, mean bilirubin levels were 169 μmol/L (range: 59-451 μmol/L). Hypoglycemia is a common indicator of severe hepatic dysfunction in AFLP, resulting from impaired hepatic glycogenolysis [18]; it was observed in 50% of our cases.

Early in AFLP, PT and coagulation factors may be normal. However, disseminated intravascular coagulation (DIC) can occur even in the initial phase, serving as a key diagnostic feature of AFLP and reported in 81% of cases [18]. In our study, 66% of patients had PT <70%, thrombocytopenia was noted in 58%, and DIC in 8% (one patient). Furthermore, 75% of our patients developed severe coagulation disorders requiring transfusion of packed red blood cells, fresh frozen plasma, and platelets. Leukocytosis, defined as a white blood cell count >11,000/mm^3^, is a hallmark of AFLP and a component of the Swansea criteria. This neutrophil-predominant leukocytosis occurs in 88% of AFLP patients, even in the absence of infection. In our study, 91% of cases exhibited leukocytosis >11,000/mm^3^.

Acute kidney injury (AKI) is reported in 50-80% of patients with acute liver failure [18]. Our findings corroborated this, with 58.33% of our patients presenting with AKI, defined as creatinine >16.9 g/L (150 μmol/L). Radiologic studies offer limited diagnostic utility in AFLP due to their low sensitivity and specificity. Abdominal ultrasound may reveal ascites or increased hepatic echogenicity [6]. In our study, serial ultrasounds detected ascites in 41% of patients and hepatic steatosis in 25%.

AFLP frequently leads to hemorrhagic complications due to associated coagulopathy, exacerbating common causes of bleeding such as uterine atony, surgical site hemorrhage, and perineal or cervical lacerations [6]. In our study, two patients experienced uterine atony, and one of them presented a cervical laceration. Patients with AFLP have a higher likelihood of requiring blood transfusions, with up to 65% needing transfusions during hospitalization [6]. In our cohort, 41% of patients required blood product transfusions. Additionally, severe obstetric hemorrhage may necessitate surgical re-exploration. Renal failure is another frequent complication, occurring in 50-80% of AFLP patients and ranging from mild AKI to severe renal failure requiring hemodialysis [18]. Our study confirms this, with seven patients exhibiting elevated creatinine levels and two requiring hemodialysis. Encephalopathy is a serious complication of AFLP, signifying acute liver failure and posing a risk of rapid progression to seizures and coma [6]. Cerebral edema and increased intracranial pressure significantly increase morbidity and mortality. AFLP is also associated with an increased risk of infections, including sepsis, pneumonia, urinary tract infections, and peritonitis [6]. In our study, three patients developed urinary tract infections, one had pneumonia, and another experienced sepsis with multiorgan failure. Acute respiratory distress syndrome and pulmonary edema necessitating mechanical ventilation were observed in one patient. While pancreatitis and transient diabetes insipidus have been reported in AFLP, they were not observed in our cohort.

The cornerstone of AFLP management is prompt delivery within 24 hours of diagnosis, alongside comprehensive supportive care. The mode of delivery is determined by maternal and fetal status, GA, fetal position, and the likelihood of successful labor induction [7]. Vaginal delivery is generally preferred if tolerated by both mother and fetus to avoid worsening coagulopathy. However, cesarean section is often recommended for fetal distress and has been associated with improved fetal and maternal outcomes [7]. In our study, half of the patients had vaginal deliveries, primarily those referred from peripheral hospitals, while the other half underwent cesarean sections.

During operative delivery, strategies to manage coagulopathy include establishing large-bore IV access and administering blood transfusions (fibrinogen, fresh frozen plasma, platelets) to maintain fibrinogen >150 mg/dL, platelet count >50,000/mm^3^, and hematocrit >30% [19]. A midline vertical incision is preferred over a transverse one to reduce bleeding risk. Intensive management of liver failure complications is crucial.

While NAC is recommended for acute liver failure, its efficacy in AFLP remains unproven. Antibiotic therapy is recommended due to the high risk of infections.

Plasmapheresis has emerged as a promising treatment for severe AFLP, acting as an artificial liver by reducing mitochondrial damage from oxidative stress. Studies have shown that plasmapheresis improves liver and kidney function, aiding recovery [20]. Literature reviews report favorable outcomes, with recovery in 50 of 53 patients in one study and 37 of 39 in another [20]. However, none of our patients received this therapy.

It is a retrospective study that limits the ability to control for all confounding variables and introduces the potential for information bias due to reliance on existing medical records.

## Conclusions

Both the existing literature and our service's protocol prioritize immediate fetal delivery as the definitive intervention to halt disease progression. While the literature explores adjunctive therapies such as NAC and plasmapheresis, the core focus remains on meticulous supportive care to address the numerous complications arising from liver failure.
